# 3-[(Methyl­carbamo­yl)amino]-1*H*-isoindolium chloride

**DOI:** 10.1107/S1600536809003699

**Published:** 2009-02-04

**Authors:** Bushra Maliha, Muhammad Ilyas Tariq, M. Nawaz Tahir, Ishtiaq Hussain, Hamid Latif Siddiqui

**Affiliations:** a University of the Punjab, Institute of Chemistry, Lahore 54590, Pakistan; b University of Sargodha, Department of Chemistry, Sargodha, Pakistan; c University of Sargodha, Department of Physics, Sargodha, Pakistan; d University of the Punjab, Institute of Chemistry, Lahore-54590, Pakistan

## Abstract

The title compound, C_10_H_12_N_3_O^+^·Cl^−^, is a derivative of *o*-phthaldehyde and methyl­thio­urea. The mol­ecules form dimers through intra- and inter­molecular N—H⋯O hydrogen bonds. The dimers are further linked into chains through one C—H⋯Cl and two N—H⋯Cl hydrogen bonds.

## Related literature

For applications of iminium salts, see: Page *et al.* (2008 ▶); Skalkos *et al.* (1994 ▶) Tariq *et al.* (2008 ▶). For the formation of derivatives of *o*-phthaldehyde with different ureas, see: Maliha, Tariq, Tahir, Hussain & Ali (2009 ▶); Maliha, Tariq, Tahir, Hussain & Siddiqui (2009 ▶); Maliha *et al.* (2008 ▶). For a related structure, see: Arfan *et al.* (2008 ▶).
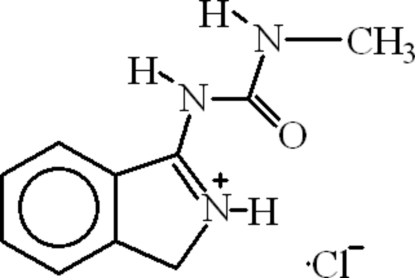


## Experimental

### 

#### Crystal data

C_10_H_12_N_3_O^+^·Cl^−^
*M* *_r_* = 225.68
Triclinic, 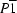

*a* = 7.1171 (5) Å
*b* = 7.7900 (6) Å
*c* = 10.3033 (8) Å
α = 89.484 (3)°
β = 69.997 (2)°
γ = 74.613 (4)°
*V* = 515.43 (7) Å^3^
*Z* = 2
Mo *K*α radiation
μ = 0.35 mm^−1^
*T* = 296 (2) K
0.30 × 0.10 × 0.06 mm

#### Data collection

Bruker Kappa APEXII CCD diffractometer
Absorption correction: multi-scan (*SADABS*; Bruker, 2005 ▶) *T*_min_ = 0.982, *T*_max_ = 0.989
8853 measured reflections
2369 independent reflections
2210 reflections with *I* > 2σ(*I*)
*R*_int_ = 0.020

#### Refinement

*R*[*F* ^2^ > 2σ(*F* ^2^)] = 0.032
*wR*(*F* ^2^) = 0.101
*S* = 1.01
2369 reflections
146 parameters
H atoms treated by a mixture of independent and constrained refinement
Δρ_max_ = 0.69 e Å^−3^
Δρ_min_ = −0.22 e Å^−3^

### 

Data collection: *APEX2* (Bruker, 2007 ▶); cell refinement: *SAINT* (Bruker, 2007 ▶); data reduction: *SAINT*; program(s) used to solve structure: *SHELXS97* (Sheldrick, 2008 ▶); program(s) used to refine structure: *SHELXL97* (Sheldrick, 2008 ▶); molecular graphics: *ORTEP-3 for Windows* (Farrugia, 1997 ▶) and *PLATON* (Spek, 2003 ▶); software used to prepare material for publication: *WinGX* (Farrugia, 1999 ▶) and *PLATON*.

## Supplementary Material

Crystal structure: contains datablocks global, I. DOI: 10.1107/S1600536809003699/ez2156sup1.cif

Structure factors: contains datablocks I. DOI: 10.1107/S1600536809003699/ez2156Isup2.hkl

Additional supplementary materials:  crystallographic information; 3D view; checkCIF report

## Figures and Tables

**Table 1:** Hydrogen-bond geometry (Å, °)

*D*—H⋯*A*	*D*—H	H⋯*A*	*D*⋯*A*	*D*—H⋯*A*
N1—H1N⋯O1	0.81 (2)	2.22 (2)	2.7097 (16)	119.9 (18)
N1—H1N⋯O1^i^	0.81 (2)	2.15 (2)	2.8760 (17)	150 (2)
N2—H2N⋯Cl1^ii^	0.90 (2)	2.23 (2)	3.0969 (13)	160.5 (18)
N3—H3N⋯Cl1^ii^	0.86 (2)	2.40 (2)	3.2082 (13)	157.0 (17)
C1—H1*B*⋯Cl1^iii^	0.97	2.74	3.6755 (16)	162

Symmetry codes: (i) 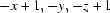; (ii) 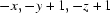; (iii) 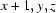.

**Fig. 1.**
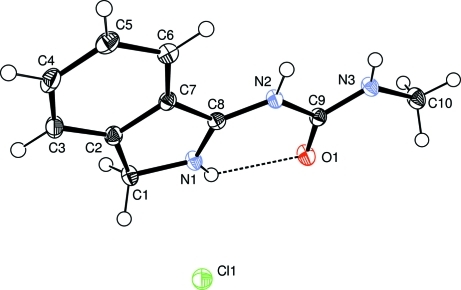
The title compound (I) with displacement ellipsoids drawn at 50% probability level. Intramolecular H-bonds are indicated by broken lines.

**Fig. 2.**
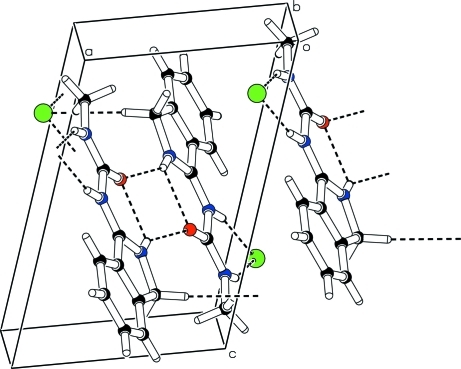
The packing of I (PLATON: Spek, 2003) showing the dimers and H-bonds to the chlorine anion. H-bonds are indicated by broken lines.

## supplementary crystallographic information

### Comment

Iminium salts are a class of organic compounds acting as reactive intermediates. These are being extensively synthesized and further reacted in an efficient and convenient manner for the preparation of multidrug-resistance reversal agents and pesticides and for metal complexation to form new photosensitizers (Page *et al.*, 2008; Skalkos *et al.*, 1994; Tariq *et al.*, 2008). The present paper relates to the continuation of our studies regarding the formation of derivatives of *O*-phthaldehyde with different ureas (Maliha *et al.*, 2008; Maliha, Tariq, Tahir, Hussain & Ali, 2009; Maliha, Tariq, Tahir, Hussain & Siddiqui, 2009).

The molecule of the title compound (I; Fig 1), is almost planar. It has three N—H bonds in the asymmetric unit. The N—H of the pyrrole ring forms an intramolecular as well as an intermolecular N–H···O H-bond. The title compound forms a dimer (Fig 2) with a central four membered O···H···O···H unit. The other two N–H groups in the methyl urea moiety are involved in intermolecular H-bonding with the Cl^-^. The Cl^-^ also forms H-bonds with the methylene group of the pyrrole ring. Consequently, the chlorine anion makes three H-bonds. Similar Cl^-^ bonding behavior has been reported by Arfan *et al.* (2008). No strong π interactions are observed.

### Experimental

*O*-phthaldehyde (200 mmol), methylthiourea (200 mmol) and a few drops of 2*M* HCl were mixed and ground in mortar and pestle. The product obtained was washed sequentially with hexane, ether, ethanol and water. The precipitate was dried and recrystallized from a mixture of methanol:acetone (9:1), by slow evaporation at room temperature.

### Crystal data

**Table:**

C_10_H_12_N_3_O^+^·Cl^−^	*Z* = 2
*M**_r_* = 225.68	*F*(000) = 236
Triclinic, *P*1	*D*_x_ = 1.454 Mg m^−^^3^
Hall symbol: -P 1	Mo *K*α radiation, λ = 0.71073 Å
*a* = 7.1171 (5) Å	Cell parameters from 2369 reflections
*b* = 7.7900 (6) Å	θ = 3.1–27.5°
*c* = 10.3033 (8) Å	µ = 0.35 mm^−^^1^
α = 89.484 (3)°	*T* = 296 K
β = 69.997 (2)°	Needle, light yellow
γ = 74.613 (4)°	0.30 × 0.10 × 0.06 mm
*V* = 515.43 (7) Å^3^	

### Data collection

**Table:**

Bruker Kappa APEXII CCD diffractometer	2369 independent reflections
Radiation source: fine-focus sealed tube	2210 reflections with *I* > 2σ(*I*)
graphite	*R*_int_ = 0.020
Detector resolution: 7.50 pixels mm^-1^	θ_max_ = 27.5°, θ_min_ = 3.1°
ω scans	*h* = −9→9
Absorption correction: multi-scan (*SADABS*; Bruker, 2005)	*k* = −10→10
*T*_min_ = 0.982, *T*_max_ = 0.989	*l* = −13→13
8853 measured reflections	

### Refinement

**Table:**

Refinement on *F*^2^	Primary atom site location: structure-invariant direct methods
Least-squares matrix: full	Secondary atom site location: difference Fourier map
*R*[*F*^2^ > 2σ(*F*^2^)] = 0.032	Hydrogen site location: mixed
*wR*(*F*^2^) = 0.101	H atoms treated by a mixture of independent and constrained refinement
*S* = 1.00	*w* = 1/[σ^2^(*F*_o_^2^) + (0.0646*P*)^2^ + 0.2853*P*] where *P* = (*F*_o_^2^ + 2*F*_c_^2^)/3
2369 reflections	(Δ/σ)_max_ < 0.001
146 parameters	Δρ_max_ = 0.69 e Å^−^^3^
0 restraints	Δρ_min_ = −0.21 e Å^−^^3^

### Special details

**Table:**

Geometry. Bond distances, angles etc. have been calculated using the rounded fractional coordinates. All su's are estimated from the variances of the (full) variance-covariance matrix. The cell esds are taken into account in the estimation of distances, angles and torsion angles
Refinement. Refinement of *F*^2^ against ALL reflections. The weighted *R*-factor *wR* and goodness of fit *S* are based on *F*^2^, conventional *R*-factors *R* are based on *F*, with *F* set to zero for negative *F*^2^. The threshold expression of *F*^2^ > σ(*F*^2^) is used only for calculating *R*-factors(gt) *etc*. and is not relevant to the choice of reflections for refinement. *R*-factors based on *F*^2^ are statistically about twice as large as those based on *F*, and *R*- factors based on ALL data will be even larger.

### Fractional atomic coordinates and isotropic or equivalent isotropic displacement parameters (Å^2^)

**Table:**

	*x*	*y*	*z*	*U*_iso_*/*U*_eq_	
O1	0.29475 (16)	0.09740 (13)	0.60677 (11)	0.0195 (3)	
N1	0.46180 (18)	0.25619 (17)	0.37719 (12)	0.0161 (3)	
N2	0.19866 (18)	0.40058 (16)	0.58681 (12)	0.0155 (3)	
N3	0.05849 (19)	0.28726 (17)	0.79177 (13)	0.0177 (3)	
C1	0.5740 (2)	0.29979 (19)	0.23859 (15)	0.0175 (4)	
C2	0.4836 (2)	0.49861 (19)	0.24873 (15)	0.0165 (4)	
C3	0.5278 (2)	0.6215 (2)	0.15233 (15)	0.0201 (4)	
C4	0.4209 (2)	0.8007 (2)	0.19298 (17)	0.0222 (4)	
C5	0.2750 (2)	0.8576 (2)	0.32626 (17)	0.0214 (4)	
C6	0.2298 (2)	0.73562 (19)	0.42329 (15)	0.0183 (4)	
C7	0.3359 (2)	0.55629 (19)	0.38103 (14)	0.0149 (4)	
C8	0.3263 (2)	0.39698 (18)	0.45638 (14)	0.0147 (3)	
C9	0.1900 (2)	0.24711 (19)	0.66151 (14)	0.0155 (3)	
C10	0.0312 (3)	0.1475 (2)	0.88541 (15)	0.0218 (4)	
Cl1	0.11104 (5)	0.28646 (4)	0.19897 (3)	0.0186 (1)	
H1A	0.54729	0.23997	0.16735	0.0210*	
H1B	0.72287	0.26795	0.21941	0.0210*	
H1N	0.487 (3)	0.156 (3)	0.401 (2)	0.0194*	
H2N	0.113 (3)	0.507 (3)	0.631 (2)	0.0186*	
H3	0.62547	0.58510	0.06360	0.0242*	
H3N	−0.007 (3)	0.397 (3)	0.819 (2)	0.0212*	
H4	0.44729	0.88501	0.12976	0.0266*	
H5	0.20729	0.97864	0.35036	0.0256*	
H6	0.13317	0.77212	0.51234	0.0220*	
H10A	0.01212	0.04982	0.83996	0.0327*	
H10B	−0.08889	0.19448	0.96722	0.0327*	
H10C	0.15238	0.10608	0.91081	0.0327*	

### Atomic displacement parameters (Å^2^)

**Table:**

	*U*^11^	*U*^22^	*U*^33^	*U*^12^	*U*^13^	*U*^23^
O1	0.0212 (5)	0.0138 (5)	0.0177 (5)	−0.0003 (4)	−0.0033 (4)	0.0024 (4)
N1	0.0167 (5)	0.0144 (6)	0.0145 (6)	−0.0019 (4)	−0.0039 (4)	0.0029 (4)
N2	0.0166 (5)	0.0126 (6)	0.0143 (6)	−0.0020 (4)	−0.0032 (4)	0.0016 (4)
N3	0.0208 (6)	0.0145 (6)	0.0145 (6)	−0.0031 (5)	−0.0036 (5)	0.0021 (4)
C1	0.0162 (6)	0.0182 (7)	0.0149 (6)	−0.0030 (5)	−0.0029 (5)	0.0021 (5)
C2	0.0146 (6)	0.0192 (7)	0.0165 (7)	−0.0058 (5)	−0.0057 (5)	0.0024 (5)
C3	0.0193 (7)	0.0251 (8)	0.0169 (7)	−0.0097 (6)	−0.0049 (5)	0.0049 (6)
C4	0.0251 (7)	0.0221 (8)	0.0249 (7)	−0.0130 (6)	−0.0111 (6)	0.0101 (6)
C5	0.0245 (7)	0.0158 (7)	0.0264 (8)	−0.0074 (6)	−0.0109 (6)	0.0047 (6)
C6	0.0181 (6)	0.0176 (7)	0.0200 (7)	−0.0053 (5)	−0.0074 (5)	0.0016 (5)
C7	0.0142 (6)	0.0160 (6)	0.0164 (7)	−0.0055 (5)	−0.0067 (5)	0.0037 (5)
C8	0.0140 (6)	0.0156 (6)	0.0158 (6)	−0.0040 (5)	−0.0071 (5)	0.0018 (5)
C9	0.0160 (6)	0.0156 (6)	0.0155 (6)	−0.0048 (5)	−0.0062 (5)	0.0033 (5)
C10	0.0280 (7)	0.0197 (7)	0.0156 (7)	−0.0068 (6)	−0.0050 (6)	0.0060 (5)
Cl1	0.0195 (2)	0.0144 (2)	0.0192 (2)	−0.0041 (1)	−0.0040 (1)	0.0002 (1)

### Geometric parameters (Å, °)

**Table:**

O1—C9	1.2234 (17)	C4—C5	1.397 (2)
N1—C1	1.4654 (19)	C5—C6	1.390 (2)
N1—C8	1.3096 (19)	C6—C7	1.392 (2)
N2—C8	1.3356 (18)	C7—C8	1.463 (2)
N2—C9	1.4197 (19)	C1—H1A	0.9700
N3—C9	1.3296 (19)	C1—H1B	0.9700
N3—C10	1.456 (2)	C3—H3	0.9300
N1—H1N	0.81 (2)	C4—H4	0.9300
N2—H2N	0.90 (2)	C5—H5	0.9300
N3—H3N	0.86 (2)	C6—H6	0.9300
C1—C2	1.501 (2)	C10—H10A	0.9600
C2—C3	1.390 (2)	C10—H10B	0.9600
C2—C7	1.395 (2)	C10—H10C	0.9600
C3—C4	1.390 (2)		
			
Cl1···C1	3.4838 (16)	C8···C7^vii^	3.457 (2)
Cl1···C2	3.6463 (16)	C9···C3^vii^	3.539 (2)
Cl1···C5^i^	3.6252 (16)	C9···C6^ii^	3.358 (2)
Cl1···N2^ii^	3.0969 (13)	C9···C4^vii^	3.513 (2)
Cl1···N3^ii^	3.2082 (13)	C9···C5^ii^	3.575 (2)
Cl1···H5^i^	2.9100	C10···C4^ii^	3.500 (3)
Cl1···H1A	2.9300	C10···C5^ii^	3.579 (3)
Cl1···H1B^iii^	2.7400	C10···C10^ix^	3.283 (2)
Cl1···H2N^ii^	2.23 (2)	C1···H10B^x^	2.9400
Cl1···H3^iv^	3.0500	C5···H10A^ii^	3.0400
Cl1···H3N^ii^	2.40 (2)	C6···H2N	2.82 (2)
Cl1···H6^ii^	2.9900	C9···H1N	2.75 (2)
Cl1···H10A^v^	3.0400	C10···H10B^ix^	3.0600
O1···O1^vi^	2.9982 (16)	C10···H10C^ix^	3.0700
O1···N1^vi^	2.8760 (17)	H1A···Cl1	2.9300
O1···N1	2.7097 (16)	H1B···Cl1^xi^	2.7400
O1···H1N^vi^	2.15 (2)	H1B···H10B^x^	2.4700
O1···H10A	2.6500	H1N···O1^vi^	2.15 (2)
O1···H1N	2.22 (2)	H1N···C9	2.75 (2)
N1···O1	2.7097 (16)	H1N···O1	2.22 (2)
N1···O1^vi^	2.8760 (17)	H2N···H6	2.4000
N2···Cl1^ii^	3.0969 (13)	H2N···H3N	2.11 (3)
N3···C5^ii^	3.436 (2)	H2N···C6	2.82 (2)
N3···C3^vii^	3.433 (2)	H2N···Cl1^ii^	2.23 (2)
N3···Cl1^ii^	3.2082 (13)	H3···Cl1^iv^	3.0500
N2···H6	2.9400	H3N···H2N	2.11 (3)
C1···Cl1	3.4838 (16)	H3N···Cl1^ii^	2.40 (2)
C2···Cl1	3.6463 (16)	H5···Cl1^viii^	2.9100
C3···N3^vii^	3.433 (2)	H6···H2N	2.4000
C3···C9^vii^	3.539 (2)	H6···N2	2.9400
C4···C9^vii^	3.513 (2)	H6···Cl1^ii^	2.9900
C4···C10^ii^	3.500 (3)	H10A···O1	2.6500
C5···N3^ii^	3.436 (2)	H10A···Cl1^v^	3.0400
C5···C9^ii^	3.575 (2)	H10A···C5^ii^	3.0400
C5···C10^ii^	3.579 (3)	H10B···C10^ix^	3.0600
C5···Cl1^viii^	3.6252 (16)	H10B···H1B^xii^	2.4700
C6···C9^ii^	3.358 (2)	H10B···C1^xii^	2.9400
C7···C8^vii^	3.457 (2)	H10C···C10^ix^	3.0700
			
C1—N1—C8	112.52 (12)	N2—C9—N3	112.47 (12)
C8—N2—C9	124.09 (12)	O1—C9—N2	121.32 (12)
C9—N3—C10	120.48 (13)	O1—C9—N3	126.20 (13)
C8—N1—H1N	125.2 (14)	N1—C1—H1A	111.00
C1—N1—H1N	122.2 (14)	N1—C1—H1B	111.00
C8—N2—H2N	118.4 (13)	C2—C1—H1A	111.00
C9—N2—H2N	117.5 (13)	C2—C1—H1B	111.00
C10—N3—H3N	121.3 (13)	H1A—C1—H1B	109.00
C9—N3—H3N	118.2 (13)	C2—C3—H3	121.00
N1—C1—C2	101.98 (12)	C4—C3—H3	121.00
C1—C2—C3	130.68 (13)	C3—C4—H4	119.00
C3—C2—C7	120.12 (13)	C5—C4—H4	119.00
C1—C2—C7	109.19 (12)	C4—C5—H5	120.00
C2—C3—C4	117.74 (14)	C6—C5—H5	120.00
C3—C4—C5	121.78 (14)	C5—C6—H6	122.00
C4—C5—C6	120.86 (14)	C7—C6—H6	122.00
C5—C6—C7	116.91 (13)	N3—C10—H10A	109.00
C2—C7—C8	106.74 (12)	N3—C10—H10B	109.00
C2—C7—C6	122.59 (13)	N3—C10—H10C	109.00
C6—C7—C8	130.66 (13)	H10A—C10—H10B	109.00
N1—C8—C7	109.54 (12)	H10A—C10—H10C	109.00
N1—C8—N2	126.82 (13)	H10B—C10—H10C	109.00
N2—C8—C7	123.63 (12)		
			
C8—N1—C1—C2	1.26 (17)	C1—C2—C7—C6	177.86 (14)
C1—N1—C8—C7	−1.95 (18)	C1—C2—C7—C8	−1.00 (17)
C1—N1—C8—N2	178.99 (15)	C3—C2—C7—C6	−1.0 (2)
C9—N2—C8—N1	−0.2 (3)	C3—C2—C7—C8	−179.83 (14)
C8—N2—C9—N3	176.55 (15)	C2—C3—C4—C5	0.6 (2)
C9—N2—C8—C7	−179.15 (14)	C3—C4—C5—C6	−0.6 (2)
C8—N2—C9—O1	−3.7 (2)	C4—C5—C6—C7	−0.1 (2)
C10—N3—C9—O1	1.4 (3)	C5—C6—C7—C8	179.48 (16)
C10—N3—C9—N2	−178.92 (15)	C5—C6—C7—C2	0.9 (2)
N1—C1—C2—C3	178.61 (16)	C2—C7—C8—N1	1.83 (18)
N1—C1—C2—C7	−0.06 (17)	C6—C7—C8—N1	−176.90 (16)
C1—C2—C3—C4	−178.36 (16)	C6—C7—C8—N2	2.2 (3)
C7—C2—C3—C4	0.2 (2)	C2—C7—C8—N2	−179.07 (15)

Symmetry codes: (i) *x*, *y*−1, *z*; (ii) −*x*, −*y*+1, −*z*+1; (iii) *x*−1, *y*, *z*; (iv) −*x*+1, −*y*+1, −*z*; (v) −*x*, −*y*, −*z*+1; (vi) −*x*+1, −*y*, −*z*+1; (vii) −*x*+1, −*y*+1, −*z*+1; (viii) *x*, *y*+1, *z*; (ix) −*x*, −*y*, −*z*+2; (x) *x*+1, *y*, *z*−1; (xi) *x*+1, *y*, *z*; (xii) *x*−1, *y*, *z*+1.

### Hydrogen-bond geometry (Å, °)

**Table:**

*D*—H···*A*	*D*—H	H···*A*	*D*···*A*	*D*—H···*A*
N1—H1N···O1	0.81 (2)	2.22 (2)	2.7097 (16)	119.9 (18)
N1—H1N···O1^vi^	0.81 (2)	2.15 (2)	2.8760 (17)	150 (2)
N2—H2N···Cl1^ii^	0.90 (2)	2.23 (2)	3.0969 (13)	160.5 (18)
N3—H3N···Cl1^ii^	0.86 (2)	2.40 (2)	3.2082 (13)	157.0 (17)
C1—H1B···Cl1^xi^	0.9700	2.7400	3.6755 (16)	162.00

Symmetry codes: (vi) −*x*+1, −*y*, −*z*+1; (ii) −*x*, −*y*+1, −*z*+1; (xi) *x*+1, *y*, *z*.
